# Antimicrobial Resistance and PFGE Molecular Typing of *Salmonella enterica* serovar Gallinarum Isolates from Chickens in South Korea from 2013 to 2018

**DOI:** 10.3390/ani12010083

**Published:** 2021-12-30

**Authors:** Jun-Feng Zhang, Ke Shang, Jong-Yeol Park, Yea-Jin Lee, Yu-Ri Choi, Sang-Won Kim, Se-Yeoun Cha, Hyung-Kwan Jang, Bai Wei, Min Kang

**Affiliations:** Department of Veterinary Infectious Diseases and Avian Diseases, College of Veterinary Medicine and Center for Poultry Diseases Control, Jeonbuk National University, Iksan 54596, Korea; jfzhang018@gmail.com (J.-F.Z.); shangke0624@gmail.com (K.S.); jyp410@naver.com (J.-Y.P.); lyj95923@naver.com (Y.-J.L.); 9cinderella7@naver.com (Y.-R.C.); sk970221@gmail.com (S.-W.K.); kshmnk@hanmail.net (S.-Y.C.); hkjang@jbnu.ac.kr (H.-K.J.); weibai116@hotmail.com (B.W.)

**Keywords:** *Salmonella enterica* serovar Gallinarum, field isolates, multi-drug resistance, pulsed-field gel electrophoresis

## Abstract

**Simple Summary:**

*Salmonella enterica* serovar Gallinarum (*S. enterica* ser. Gallinarum) is a host-specific agent of fowl typhoid (FT). This is one of the most important bacterial infections in the poultry industry in both developing and developed countries, including South Korea. The use of antimicrobial drugs is the first choice for disease control. Antimicrobials, such as β-lactams, aminoglycosides, and fluoroquinolones, are frequently used to treat FT. However, the continuous use of antimicrobial drugs has led to the emergence and persistence of antimicrobial-resistant *S. enterica* ser. Gallinarum. In this study, we analyzed the antimicrobial susceptibility and epidemiological relationship of thirty isolates of *S. enterica* ser. Gallinarum isolated from poultry farms with an FT outbreak from 2013 to 2018 in South Korea. All the isolates showed a multi-drug resistant (MDR) phenotype. This study confirmed horizontal transmission and cross-contamination between farms within the same integrated poultry company or between farms belonging to different companies. The characterization of these isolates would be helpful to develop prevention and control strategies for the MDR *S. enterica* ser. Gallinarum infection in South Korea.

**Abstract:**

Antimicrobial resistance and pulsed-field gel electrophoresis (PFGE) genotypes of collected *S. enterica* ser. Gallinarum isolates were investigated to examine the epidemiological relationship between field outbreak isolates of *S. enterica* ser. Gallinarum. Thirty *S. enterica* ser. Gallinarum isolates collected from poultry farms with FT outbreaks from 2013 to 2018 in South Korea were analyzed. All isolates were resistant to at least 3 of the 18 antimicrobials tested and exhibited an MDR phenotype. All isolates showed resistance to streptomycin, sulfisoxazole, and colistin. One isolate was resistant to 9 antimicrobials. The antimicrobial resistance profile, streptomycin-sulfisoxazole-colistin-nalidixic acid-ciprofloxacin-gentamicin (18/30, 60.0%), was the most prevalent. PFGE types were classified into 10 groups with a 100% correlation cutoff in dendrograms for 30 field isolates. The dominant PFGE types were 1 (8/30, 26.7%), 4 (7/30, 23.3%), and 9 (5/30, 16.7%). Interestingly some isolates collected from the same and different companies had the same PFGE type. We reported a high MDR rate in *S. enterica* ser. Gallinarum isolates. The present study highlights the occurrence of horizontal spread and cyclic contamination of MDR *S. enterica* ser. Gallinarum within the same company. Furthermore, we showed cross-contamination between different companies. The characterization of these isolates would be helpful in the development of prevention and control strategies for MDR *S. enterica* ser. Gallinarum infection in South Korea.

## 1. Introduction

*Salmonella enterica* serovar Gallinarum (*S. enterica* ser. Gallinarum) is the causative agent of fowl typhoid (FT). This is a disease characterized by severe hepatomegaly and splenomegaly accompanied by a liver with bronzing aspects, anemia, and septicemia, with mortality rates of up to 80% in affected chickens of any age [1,2,3]. FT has been almost eradicated from commercial poultry in many developed countries, including North American, Canada, Australia, Japan, and most European countries. This was completed by isolating and removing infected flocks, as well as implementing biosecurity and hygiene measures. Nonetheless, FT remains a significant economic problem in many parts of the world, such as Africa, Asia, and Central and South America [4,5].

After the first occurrence of FT in the field in 1992, it spread quickly throughout South Korea because the majority of the population was comprised of brown layers, which are more vulnerable to FT [6]. Nevertheless, producers would not replace brown layers with white chickens due to the preferences of Korean customers. Since then, FT has become one of the most serious bacterial diseases in the poultry industry. After suffering substantial economic losses due to this disease, South Korea opted to implement a nationwide vaccination program with a live attenuated strain *S. enterica* ser. Gallinarum 9R (SG9R) for commercial layers in 2001, rather than an eradication policy, to reduce the economic impact of FT. Subsequently, the number of reported cases decreased significantly. However, the vaccine has many shortcomings [7,8,9]; therefore, the use of antimicrobial drugs remains the first choice for disease control.

Antimicrobials, such as β-lactams, aminoglycosides, and fluoroquinolones, are frequently used to treat systemic bacterial infections, including those caused by *S. enterica* ser. Gallinarum in commercial chicken farms in South Korea. Fluoroquinolones, in particular, are extensively utilized due to the advantages of oral administration and strong efficacy against a wide range of Gram-negative bacteria [10]. However, the continuous use of antimicrobial drugs has led to the emergence and persistence of antimicrobial-resistant *S. enterica* ser. Gallinarum [10,11,12]. In recent years, many multidrug-resistant (MDR) *S. enterica* ser. Gallinarum isolates have been reported [11,13,14].

During a disease outbreak, a detailed epidemiological investigation would directly influence measures that should be adopted for the application of a successful control plan. Pulsed-field gel electrophoresis (PFGE) is an efficient and reliable method to identify the sources of infection or reservoirs of epidemic outbreaks, to understand the clonality among isolates and to determine transmission routes [15]. PFGE works by digesting the whole DNA using restriction enzymes to yield strain-specific fragment patterns. This method is based on genomic differences between isolates. It is these differences accumulated by genetic variation that cause slight detectable differences between DNA fingerprint patterns [16].

Here, we studied the antimicrobial susceptibility of thirty isolates of *S. enterica* ser. Gallinarum isolated from poultry farms with FT outbreaks from 2013 to 2018 in South Korea. We analyzed the epidemiological relationship of these isolates to determine whether there was horizontal transmission and cross-contamination of MDR isolates between farms belonging to the same or different integrated poultry companies.

## 2. Materials and Methods

### 2.1. Bacterial Isolates

The thirty isolates of *S. enterica* ser. Gallinarum used in this study were provided by the Department of Veterinary Infectious Diseases and Avian Diseases, College of Veterinary Medicine and Center for Poultry Diseases Control, Jeonbuk National University. The thirty *S. enterica* ser. Gallinarum isolates chosen were suspected of having an epidemiological relationship. This center has long-term cooperative relationships with main poultry companies in South Korea for disease monitoring. It should be emphasized that these isolates were representative isolates selected from the samples of FT outbreaks in farms of poultry companies from 2013 to 2018. These isolates were isolated from farms belonging to eight different integrated poultry companies in South Korea (Table 1). Among these isolates, some were isolated from the same farm of the same company, some were isolated from different farms of the same company, and some were isolated from different farms from different companies. To isolate *Salmonella enterica*, aseptically separated liver samples were directly streaked onto MacConkey Agar (Difco Laboratories, Detroit, MI, USA), and the plate was incubated for 24 h at 37 °C. Presumptive *Salmonella* colonies were further tested using a *Salmonella* latex test kit (Thermo Fisher Scientific, Oxoid Ltd., Basingstoke, UK). All positive isolates were serotyped per the Kauffmann–White scheme using slide agglutination with O and H antigen-specific sera (BD Difco, Sparks, MD, USA and Denka Seiken Co., Ltd., Tokyo, Japan). *S. enterica* ser. Gallinarum isolates were confirmed by the agglutination test using *Salmonella* O antiserum group D1, the motility test and biochemical tests. The isolates were also identified using VDx^®^ Fowl Typhoid PCR (MEDIAN Diagnostics, Chuncheon, Gangwon-do, Korea). The *S. enterica* ser. Gallinarum live vaccine Nobilis SG9R (Intervet International, Boxmeer, The Netherlands) was used as a reference strain. All isolates were stored in Luria-Bertani (LB) medium containing 20% glycerol at −70 °C.

### 2.2. Antimicrobial Susceptibility Test

The KRNV5F Sensititre panel (TREK Diagnostic Systems, Incheon, Korea) was used to determine minimum inhibitory concentrations (MICs) according to the manufacturer’s instructions [17]. The antimicrobials used for MIC were amoxicillin/clavulanic acid (AUG2, 2/1–32/16 μg/mL), ampicillin (AMP, 2–64 μg/mL), cefoxitin (FOX, 1–32 μg/mL), ceftazidime (TAZ, 1–16 μg/mL), ceftiofur (XNL, 0.5–8 μg/mL), cefepime (FEP, 0.25–16 μg/mL), chloramphenicol (CHL, 2–64 μg/ mL), ciprofloxacin (CIP, 0.12–16 μg/mL), colistin (COL, 2–16 μg/mL), florfenicol (FFC, 2–64 μg/mL), gentamicin (GEN, 1–64 μg/mL), Kanamycin (K, 4–256 μg/mL), meropenem (MERO, 0.25–4 μg/mL), nalidixic acid (NAL, 2–128 μg/mL), sulfisoxazole (FIS, 16–256 μg/mL), streptomycin (STR, 16–128 μg/mL), tetracycline (TET, 2–128 μg/mL), and trimethoprim/sulfamethoxazole (SXT, 0.12/2.38–4/76 μg/mL). The quality control strain that was used was *Escherichia coli* ATCC 25922. The interpretive categories—susceptible, intermediate, or resistant—were used according to the Clinical and Laboratory Standard Institute (CLSI) guidelines [18], except for colistin, where an MIC value ≥4 μg/mL (resistant) was used [19]. *Salmonella* isolates, resistant to three or more antimicrobial classes, were defined as multidrug resistant (MDR).

### 2.3. PFGE and BioNumerics Analysis

*S. enterica* ser. Gallinarum isolates (*n* = 31, 30 field isolates and vaccine strain SG9R) were analyzed using PFGE, with some modifications, according to the Centers for Disease Control and Prevention procedures, as previously described [20]. In brief, a single colony of each isolate was streaked onto a MacConkey agar plate and incubated at 37 °C overnight. The bacteria were then suspended in PBS at a concentration of 0.6–0.8 optical density (OD). Genomic DNA samples were digested with 50U of *Xba*I (Thermo Fisher Scientific, Inchon, Korea) at 37 °C for 3 h after extraction with 1% SDS and 1 mg/mL proteinase K (Biosesang, Seoul, Korea). Using a CHEF-DR^@^ electrophoresis equipment (Bio-Rad, Hercules, CA, USA), the digested DNA was separated by electrophoresis in 0.5× TBE buffer at 14 °C for 18 h at a gradient of 6 V/cm, with a pulse angle of 120°. The pulse time was gradually increased from 2.16 to 63.8 s. The fragment sizes were determined using fragments from the *Salmonella enterica* serovar Braenderup H9812 reference standard (ATCC^®^ BAA-664™). After electrophoresis, gels were stained using ethidium bromide solution. Then, the band patterns were photographed on a UV transilluminator (Bio Doc-It Imaging System, Upland, CA, USA). The gel images were analyzed by BioNumerics (version 5.10 for Windows, Applied Maths, Sint-Martens-Latem, Belgium). The dendrogram tree was constructed using Unweighted Pair Group Method with Arithmetic Mean (UPGMA) with Dice coefficient at an optimization setting of 1% and a position tolerance setting of 1%. The band of 100% similarity was regarded as the same PFGE type.

## 3. Results

### 3.1. Antimicrobial Susceptibility Test

The antimicrobial susceptibility test results of *S. enterica* ser. Gallinarum isolates is shown in Table 2. All isolates showed resistance to STR, FIS, and COL. The resistance rates to NAL, CIP, and GEN were 96.7%, 90.0%, and 66.7%, respectively. A 3.3% (1/30) resistance rate was observed in CHL, AMP, TET, and FFC. No isolates were resistant to the eight antimicrobials, including AUG2, FEP, FOX, SXT, TAZ, XNL, MERO, and K. Five antimicrobial resistance profiles were observed among the isolates. The antimicrobial resistance profile, STR-FIS-COL-NAL-CIP-GEN (18/30, 60.0%), was the most prevalent, followed by STR-FIS-COL-NAL-CIP (8/30, 27.0%). In addition, one isolate had a nine antimicrobial resistance pattern of STR-FIS-COL-NAL-CIP-CHL-AMP-TET-FFC. The distribution of MDR by year is shown in Table 3. All isolates were classified as MDR because they were resistant to ≥3 classes of antimicrobials. One isolate, A16-OTH-010 (3.3%), was resistant to eight antimicrobial classes. There were 26 isolates (86.7%) resistant to five antimicrobial classes. Two isolates were resistant to four antimicrobial classes. One isolate, A16-MRA-002 (3.3%), was resistant to three antimicrobial classes.

### 3.2. PFGE and Bionomics Analysis

**Figure 1:**
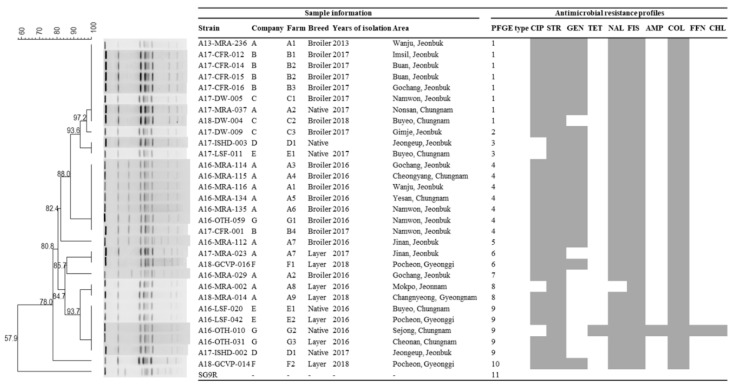
A dendrogram based on the *Xba*I-pulsed field gel electrophoresis (PFGE) profiles of *S. enterica* ser. Gallinarum isolates from chicken. Corresponding antimicrobial susceptibility patterns to the 18 indicated antimicrobials. Gray squares represent resistance.

Thirty-one isolates of *S. enterica* ser. Gallinarum, including thirty field isolates collected from 2013–2018 and a vaccine strain (SG9R), were analyzed by PFGE after DNA digestion with *Xba*I (Figure 1). Eleven different PFGE types (1 to 11), exhibiting 100% similarity, were produced by enzyme digestion. The vaccine strain (SG9R) notably showed a different type (type 11). The dominant PFGE types were type 1 (8/30, 26.7%), 4 (7/30, 23.3%), and 9 (5/30, 16.7%). Isolates of the same PFGE type were observed in different companies (type 1 in companies A, B, and C; type 3 in companies D and E; type 4 in companies A, B, and G; type 6 in companies A and F; type 9 in companies D, E, and G). Conversely, isolates of different PFGE types were observed in the same companies (types 1, 4, 5, 6, 7 and 8 in company A; types 1 and 4 in company B; types 1 and 2 in company C; types 3 and 9 in companies D and E; types 6 and 10 in company F; types 4 and 9 in company G). There were five PFGE types in broiler chickens, namely types 1, 2, 4, 5, and 7; type 1 was the dominant type. There were four types of PFGE in layers, 6, 8, 9, and 10. There were three types of PFGE in native chickens, 1, 3, and 9. There was only one isolate taken from 2013, which had the PFGE type 1. The PFGE types from isolates in 2016 included 4, 5, 7, 8, and 9. The PFGE types from isolates in 2017 included 1, 2, 3, 4, 6, and 9. The PFGE types of isolates from 2018 included 1, 6, 8, and 10.

## 4. Discussion

In this study, thirty isolates from poultry farms with FT outbreaks from 2013 to 2018 were used. We used antimicrobial susceptibility tests to understand the drug resistance and PFGE to evaluate the genetic relationship of these isolates. We aimed to confirm whether there was horizontal transmission and cross-contamination between farms within the same or different companies.

A previous study has reported that the STR resistance rates of *S. enterica* ser. Gallinarum isolates from broilers and layers between 1999 and 2004 were 99.8% (55/56) and 81.8% (36/44), respectively [21]. In the present study, all isolates were STR resistant, which suggested that STR may be completely ineffective for the treatment of FT in poultry. Resistance to GEN, an aminoglycoside antibiotic such as STR, reached 64.5% in the present study. Compared with 56.6% in 2011, the GEN resistance rate has increased significantly [12]. Aminoglycosides, such as STR and GEN, are the most commonly used antimicrobials in poultry [10,22]. Therefore, this increased resistance may be related to their constant use. In addition, the use of antimicrobials as growth promoters remains common, which may have indirectly promoted the emergence of resistant strains of *Salmonella*.

It is noteworthy that the resistance to COL has also reached 100% in the present study. From 2014–2017, COL sales increased sharply, which suggests that the increased resistance of COL may be a consequence of its heavy use [11]. Infections caused by MDR gram-negative bacteria have increased dramatically, and polymyxins are often the only active antibiotics available [18]. In Europe, plasmid-mediated COL resistance in *Enterobacteriaceae* has spread widely in avian and pig farms. This has necessitated immediate international action to restrict or ban COL use in agriculture to prevent further resistance spread [23]. *Mobilized colistin resistance* (*mcr*) genes are plasmid-borne genes that confer resistance to COL. To date, *mcr*-1 to *mcr*-9 genes have been reported [24]. However, none of these genes were detected in our isolates (personal communication). Therefore, other resistance genes or mechanisms related to COL need to be studied. COL resistance can be transmitted to humans through the food chain, posing a threat to human health. Hence, it must be continuously monitored.

The resistance rate of *S. enterica* ser. Gallinarum isolates to CIP has increased from 0% in 1995 to 89.1% in 2002 [12]. The CIP resistance rate of our isolates reached 90%, which was consistent with the 2002 rate. Enrofloxacin (ENR) is metabolized to CIP; therefore, excessive use of ENR will correspondingly increase CIP resistance. The Food and Drug Administration (FDA) banned the use of ENR in the USA in 2005; however, it was not banned in chickens until May 2017 in South Korea [11,25]. Prior to this, ENR was continuously applied to poultry through drinking water, which may be the cause of increased resistance to CIP.

Between 1995 and 2001, 16.2% (22/136) of the isolates were MDR [12]. Of the isolates isolated between 2002 and 2007, 61.0% were MDR [10]. Of the isolates isolated in 2018, 60.7% (17/128) were MDR [11]. In this study, all isolates isolated between 2013 and 2018 were MDR, which suggested that the isolation of MDR isolate has become common and reflects the severity of the resistance of *S. enterica* ser. Gallinarum in Korea. This poses even greater hurdles for FT prevention and control in the poultry industry. The emergence of MDR isolates is usually caused by excessive and unreasonable simultaneous use of multiple antimicrobials. Here, it is important to note that there was one isolate that was resistant to nine different antimicrobials, which brings greater challenges to the use of antimicrobial treatment. Therefore, the monitoring of MDR isolates must be strengthened. Otherwise, more resistant super bacteria may appear. The universality and severity of MDR isolates once again emphasize the importance of the rational use of antimicrobials.

Many bacteria, including various *Salmonella* serotypes, have been successfully tested using PFGE. This genetic analysis can assess the genetic diversity of the entire genome. It is the gold standard method due to its accuracy and reproducibility [26,27,28,29]. First, isolates collected from different farms belonging to the same integrated company had the same PFGE type (for example, type 1 in B1, B2, and B3 farms belonging to company B; type 4 in A1, A3, A4, A5 and A6 farms belonging to company A). This indicated that the horizontal spread of MDR isolates existed between farms within the same company. This could be because the farms that belong to the same company share resources, including breeders, trucks for transport, veterinarians, chicks, and feed. Second, isolates collected from different farms belonging to different integrated companies had the same PFGE type (for example, type 1 in companies A, B, and C; type 4 in companies A, B, and G; type 9 in companies D, E, and G). This indicated that cross-contamination exists between different companies. Migratory birds may play a role in the dispersal of pathogenic and antimicrobial-resistant *Salmonella* in poultry [30]. Therefore, we hypothesize that migratory birds may be one of the causes of cross-contamination between companies. Third, some isolates had the same PFGE type and antimicrobial resistance profile (for example, type 1 isolates (A13-MRA-236, A17-CFR-012, A17-CFR-014, A17-CFR-015, A17-CFR-016, A17-DW-005, A17-MRA-037) with CIP-STR-GEN-NAL-FIS-COL; type 4 isolates (A16-MRA-114, A16-MRA-115, A16-MRA-116, A16-MRA-134, A16-MRA-135, A16-OTH-059, and A17-CFR-001 with CIP-STR-GEN-NAL-FIS-COL)). This further indicated that some isolates might come from the same clone.

Interestingly, the PFGE types (1, 2, 4, 5, and 7) of the isolates collected from broilers were different from the PFGE types (6, 8, 9, and 10) of the isolates collected from the layers. This indicated that there were different isolate genotypes infecting broilers and layers. One explanation might be that different breeds of chickens have different susceptibilities to *S. enterica* ser. Gallinarum [31]. Additionally, layers are older and have a longer exposure period than broilers. Their treatment regimens often differ as well. Further research and analysis are needed to explain this result.

Whole-genome sequencing (WGS) is an important tool for the analysis of resistant bacteria. WGS technology can be used to analyze drug resistance genes and their mutations. In addition to known drug resistance mechanisms, it can also be used to predict unknown potential drug resistance mechanisms [32,33]. To deeply study the resistance genes and resistance mechanisms of these currently isolated *S. enterica* ser. Gallinarum isolates, WGS has been listed as a further plan.

## 5. Conclusions

We reported a high MDR rate in *S. enterica* ser. Gallinarum isolates from farms of poultry companies from 2013 to 2018 in South Korea. The present study highlights the horizontal spread of MDR *S. enterica* ser. Gallinarum within and between the same and different companies. We believe the characterization of these isolates will be helpful in the development of prevention and control strategies.

## Figures and Tables

**Table 1:** Isolates of *S. enterica* ser. Gallinarum used in the present study.

No.	Isolate	Company	Farm	Source	Breed	Age (d)	Year	Case History	Location (City and Province)
1	A13-MRA-236	A	A1	Liver	Broiler	8	2013	-	Wanju, Jeonbuk
2	A16-LSF-020	E	E1	Liver	Native	34	2016	-	Buyeo, Chungnam
3	A16-LSF-042	E	E2	Liver	Layer	360	2016	FT ^a^ symptoms	Pocheon, Gyeonggi
4	A16-MRA-002	A	A8	Liver	Layer	179	2016	-	Mokpo, Jeonnam
5	A16-MRA-029	A	A2	Liver	Broiler	25	2016	FT symptoms	Gochang, Jeonbuk
6	A16-MRA-112	A	A7	Liver	Broiler	8	2016	1240 bird deaths in 4 d	Jinan, Jeonbuk
7	A16-MRA-114	A	A3	Liver	Broiler	9	2016	Death increased suddenly from 5 d of age	Gochang, Jeonbuk
8	A16-MRA-115	A	A4	Liver	Broiler	7	2016	1000 bird deaths within a week	Cheongyang, Chungnam
9	A16-MRA-116	A	A1	Liver	Broiler	8	2016	FT symptoms	Wanju, Jeonbuk
10	A16-MRA-134	A	A5	Liver	Broiler	28	2016	FT symptoms	Yesan, Chungnam
11	A16-MRA-135	A	A6	Liver	Broiler	9	2016	870 bird deaths/d	Namwon, Jeonbuk
12	A16-OTH-010	G	G2	Liver	Native	70	2016	Cumulative 2000 bird deaths	Sejong, Chungnam
13	A16-OTH-031	G	G3	Liver	Layer	245	2016	From 28 weeks of old, 10–20 deaths/d	Cheonan, Chungnam
14	A16-OTH-059	G	G1	Liver	Broiler	9	2016	1000 bird deaths in 8 days	Namwon, Jeonbuk
15	A17-CFR-001	B	B4	Liver	Broiler	9	2017	FT symptoms (Enrofloxacin administration)	Namwon, Jeonbuk
16	A17-ISHD-002	D	D1	Liver	Native	44	2017	10 bird deaths/d	Jeongeup, Jeonbuk
17	A17-MRA-023	A	A7	Liver	Layer	245	2017	-	Jinan, Jeonbuk
18	A17-ISHD-003	D	D1	Liver	Native	57	2017	50–60 bird deaths (Enrofloxacin administration)	Jeongeup, Jeonbuk
19	A17-MRA-037	A	A2	Liver	Native	95	2017	Bronze liver, enlarged spleen, and FT symptoms	Nonsan, Chungnam
20	A17-LSF-011	E	E1	Liver	Native	49	2017	229 bird deaths in 5 d	Buyeo, Chungnam
21	A17-CFR-012	B	B1	Liver	Broiler	18	2017	3.1% mortality, 10 bird deaths/d	Imsil, Jeonbuk
22	A17-CFR-014	B	B2	Liver	Broiler	8	2017	Liver enlargement, hemorrhage, and necrosis	Buan, Jeonbuk
23	A17-DW-005	C	C1	Liver	Broiler	10	2017	4% mortality within one week	Namwon, Jeonbuk
24	A17-DW-009	C	C3	Liver	Broiler	9	2017	1300 bird deaths in 9 d	Gimje, Jeonbuk
25	A17-CFR-015	B	B2	Liver	Broiler	8	2017	50 bird deaths/d	Buan, Jeonbuk
26	A17-CFR-016	B	B3	Liver	Broiler	7	2017	Death increased from 7 d old	Gochang, Jeonbuk
27	A18-DW-004	C	C2	Liver	Broiler	10	2018	FT symptoms	Buyeo, Chungnam
28	A18-GCVP-014	F	F2	Liver	Layer	457	2018	0.28% mortality within one week; FT symptoms	Pocheon, Gyeonggi
29	A18-GCVP-016	F	F1	Liver	Layer	35	2018	-	Pocheon, Gyeonggi
30	A18-MRA-014	A	A9	Liver	Layer	237	2018	-	Changnyeong, Gyeongnam

-, no information. ^a^ FT: fowl typhoid.

**Table 2:** Antimicrobial resistance patterns of *S. enterica* ser. Gallinarum isolates (*n* = 30).

Year	Isolates	Antimicrobial Resistance Profile										
		Pattern No.	Antimicrobials ^a^									
2013 (*n* = 1)	A13-MRA-236	1	STR	FIS	COL	NAL	CIP	GEN				
2016 (*n* = 13)	A16-MRA-112	1	STR	FIS	COL	NAL	CIP	GEN				
	A16-MRA-114	1	STR	FIS	COL	NAL	CIP	GEN				
	A16-MRA-115	1	STR	FIS	COL	NAL	CIP	GEN				
	A16-MRA-116	1	STR	FIS	COL	NAL	CIP	GEN				
	A16-MRA-134	1	STR	FIS	COL	NAL	CIP	GEN				
	A16-MRA-135	1	STR	FIS	COL	NAL	CIP	GEN				
	A16-OTH-059	1	STR	FIS	COL	NAL	CIP	GEN				
	A16-LSF-020	2	STR	FIS	COL	NAL	CIP					
	A16-LSF-042	2	STR	FIS	COL	NAL	CIP					
	A16-MRA-029	2	STR	FIS	COL	NAL	CIP					
	A16-OTH-031	2	STR	FIS	COL	NAL	CIP					
	A16-MRA-002	3	STR	FIS	COL							
	A16-OTH-010	4	STR	FIS	COL	NAL	CIP		CHL	AMP	TET	FFC
2017 (*n* = 12)	A17-CFR-001	1	STR	FIS	COL	NAL	CIP	GEN				
	A17-MRA-037	1	STR	FIS	COL	NAL	CIP	GEN				
	A17-CFR-012	1	STR	FIS	COL	NAL	CIP	GEN				
	A17-CFR-014	1	STR	FIS	COL	NAL	CIP	GEN				
	A17-DW-005	1	STR	FIS	COL	NAL	CIP	GEN				
	A17-DW-009	1	STR	FIS	COL	NAL	CIP	GEN				
	A17-CFR-015	1	STR	FIS	COL	NAL	CIP	GEN				
	A17-CFR-016	1	STR	FIS	COL	NAL	CIP	GEN				
	A17-ISHD-002	2	STR	FIS	COL	NAL	CIP					
	A17-MRA-023	2	STR	FIS	COL	NAL	CIP					
	A17-ISHD-003	5	STR	FIS	COL	NAL		GEN				
	A17-LSF-011	5	STR	FIS	COL	NAL		GEN				
2018 (*n* = 4)	A18-GCVP-014	1	STR	FIS	COL	NAL	CIP	GEN				
	A18-GCVP-016	1	STR	FIS	COL	NAL	CIP	GEN				
	A18-DW-004	2	STR	FIS	COL	NAL	CIP					
	A18-MRA-014	2	STR	FIS	COL	NAL	CIP					
Total (*n* = 30)			30/30 (100%)	30/30 (100%)	30/30 (100%)	29/30 (96.7%)	27/30 (90.0%)	20/30 (66.7%)	1/30 (3.3%)	1/30 (3.3%)	1/30 (3.3%)	1/30 (3.3%)

^a^ STR, streptomycin; FIS, sulfisoxazole; COL, colistin; NAL, nalicixic acid; CIP, ciprofloxacin; GEN, gentamicin; CHL, Chloramphenicol; AMP, ampicillin; TET, tetracycline; FFC, florfenicol.

**Table 3:** Multi-drug resistance of *S. enterica* ser. Gallinarum isolates obtained from 2013 to 2018.

Antimicrobial Resistance Categories	No. of Classes	No. of Antimicrobials		No. of Isolates Shown Resistance (%)					
			2013 (*n* = 1)	2014 (*n* = 0)	2015 (*n* = 0)	2016 (*n* = 13)	2017 (*n* = 12)	2018 (*n* = 4)	Total (*n*)
AMGs-SAs-Qs-POLs-FQs-PHs-BLAs-TETs	8	9	-	-	-	1 (7.7)	-	-	1
AMGs-SAs-Qs-POLs-FQs,	5	6	1 (100)	-	-	7 (53.8)	8 (66.7)	2 (50.0)	18
	5	5	-	-	-	4 (30.8)	2 (16.7)	2 (50.0)	8
AMGs-SAs-Qs-POLs	4	5	-	-	-	-	2 (16.7)	-	2
AMGs-SAs-POLs	3	3	-	-	-	1 (7.7)	-	-	1
Total (%)	-	-	1 (100)	-	-	13 (100)	12 (100)	4 (100)	30

AMGs, aminoglycosides; SAs, sulfonamides; Qs, quinolones; POLs, polymyxins; FQs, fluoroquinolones; PHs, phenicols; BLAs, β-lactams; TETs, tetracyclines. -, no isolates.

## Data Availability

The data presented in this study are available from the corresponding author on reasonable request.
